# Neurotropic Melanoma: The Management of Localised Disease

**DOI:** 10.1155/2012/706452

**Published:** 2012-10-22

**Authors:** Jeremy Croker, Bryan Burmeister, Matthew Foote

**Affiliations:** ^1^Radiation Oncology Queensland, Liz Plummer Cancer Center, Lake and Grove Streets, Cairns, QLD 4870, Australia; ^2^Division of Cancer Services, Princess Alexandra Hospital, University of Queensland, 199 Ipswich Road Woolloongabba, QLD 4102, Australia

## Abstract

Neurotropic melanoma is a rare subtype of cutaneous malignant melanoma. Compared with conventional melanoma, it is more locally aggressive with an increased tendency for local recurrence but less likely for nodal or distant metastases. These tumours can be a diagnostic dilemma with a variety of morphological, histopathological, and immunophenotypical expressions. The often amelanotic, benign appearance may lead to treatment issues such as late presentation, diagnostic delay, misdiagnosis, insufficient surgical margins, and recurrence with resulting poor outcome. The neurotropic nature of the disease and prevalence in the head and neck region can result in perineural and neural invasion along named large nerves into the brain with resulting neuropathies. Wide local excision with adjuvant radiotherapy where indicated remains the current practice for treatment with chemotherapy predominately being reserved as a salvage treatment for patients with disseminated disease.

## 1. Introduction

Neurotropic melanoma (NM) is a rare variant of cutaneous melanoma with a high incidence of local recurrence and low rate of distant metastases [1, 2]. Appearance of these lesions is highly variable and can mimic benign lesions which create diagnosis and therapeutic difficulties [3–6]. Neurotropic melanoma often displays desmoplasia. Desmoplastic neurotropic melanoma (DNM) is a well-described neurotropic subtype of desmoplastic melanoma (DM) [7]. However, diagnostic criteria to differentiate DNM from DM are not well described [8].

Neurotropism describes direct invasion into neural structures (intraneural) or surrounding nerve (perineural) infiltration or cells exhibiting neural or nerve sheath differentiation [1, 3, 4, 7]. Neurotropism can be associated with worse prognosis in melanoma due to its propensity for deep invasion at the primary site and potential for positive margins at the excision site due to neural extension [9].

Neurotropic melanoma, DNM, and DM have higher local recurrence rates when treated with surgical excision alone [1, 4]. A systemic review of 14 studies involving DM demonstrated local recurrence rates ranging from 7 to 56% [3]. This is compared with a rate of 3% for other melanoma [9]. Regional, nodal, and distant metastatic disease rates are lower than with other forms of cutaneous melanoma [1–3, 6, 7, 10]. Long-term control of local disease is therefore paramount [1].

This paper reviews current literature regarding this rare melanoma variant, describing its defining characteristics, exploring the diagnostic challenges, current therapeutic approach, and future treatment and research directions.

## 2. Historical Perspective

First described in 1971 by Conley et al. [11], DM is a primarily dermal subtype of invasive cutaneous melanoma comprising dermal spindle cells separated by collagen fibres or fibrous stroma [5, 12]. Reed and Leonard [13] in 1979 delineated DNM as a subset of DM expressing Schwannian differentiation and neurotropic characteristics which may extend beyond the desmoplastic component. Over a 10-year study period at the Sydney Melanoma Unit, DNM represented 90 (1.3%) of the total 6791 new patients examined [12]. Desmoplastic neurotropic melanoma represents at least 30% of DM [1, 4, 5] and has been reported in up to 82% of cases in certain DM series [14].

There have been several hundred cases of NM described in series and discussion surrounds the differentiation of NM, DNM, and DM [2, 14]. Kay et al. [14] proposed the description “desmoplastic melanoma with neurotropism” for DNM as both DM and DNM were likely different phenotypes of the same tumour. Another hypothesis is that these tumours exist within a neuroectodermal tumour continuum, similar to nerve sheath tumours, as reflected in their propensity for neurotropism and their immunohistochemical profile [5, 6, 15].

Less common is NM without desmoplasia, whereby melanomas are solid spindle or epithelioid cell origin and have a nondesmoplastic growth phase but perineural or endoneurial involvement [5, 9, 16].

## 3. Clinical Considerations

### 3.1. Presentation

Due to rarity of the disease, there is limited epidemiological data or descriptions of the natural history of NM [6]. Neurotropic melanoma provides a diagnostic challenge as it presents differently to other melanomas, often as slow-growing, painless, innocuous-appearing amelanotic lesions [5–7, 12, 17], leading to misdiagnosis and/or late diagnosis with associated poorer prognosis [4, 5]. Lesions may be discrete discoid papule, nondescript plaque, and thickening or nodular and ulcerative in nature [3, 4, 7]. The benign appearance may lead to insufficient initial excision margins [7].

Lesions most often are associated with lentigo maligna (melanoma in situ) [6] but they may occur with other melanoma types [12, 18]. Macroscopically they may resemble squamous cell or basal cell carcinomas, dermatofibromas, sarcomas, cysts, or scar tissue [3, 5, 17]. Size of lesions varies from 4 to 60 mm [19]. Margins of the lesions are often ill defined and satellite lesions present [6]. The less common are “de novo” tumours in which there is no intraepidermal melanocytic component. In rare circumstances, a conventional melanoma may reoccur as a DNM [19].

Cases of NM reported in the literature have been exclusively Caucasian. Approximately two-thirds of cases are male, which is a larger percentage than with conventional melanoma [3, 4, 14]. The ratio of male to female is even higher in those who undergo radiotherapy, suggesting males have more advanced disease at presentation [4]. Presentation is most common in the seventh decade of life [4, 12, 14]. This is older for other forms of cutaneous melanoma (median age of 46 years) [4].

Over half of DNM and NM lesions are located on the head and neck [1, 3–6, 12, 14, 19, 20]. This is compared with 19% for other melanomas [4]. The location of lesions on the head or neck has been associated with poorer outcomes. Lesions on the extremities (upper more than lower limbs) are more common than on the trunk [3, 4, 17]. However, any anatomical site may be involved including oral, conjunctival, and genital sites [6]. As with other forms of cutaneous melanoma, sun exposure is a risk factor for NM and DNM, [1, 3, 4, 12, 14, 21, 22] as is fair skin [12, 20].

Nodal metastases at initial presentation or recurrence are not as common as other cutaneous melanomas, with an incidence range of 6 to 11% [4, 6, 12, 17]. There are reports of low yield from lymph node biopsy (SLNB) [7, 17]. Chen et al. [4] hypothesise that the lower occurrence of nodal metastases with DM (including DNM) may be due to their possible differing biology and natural history compared to other melanoma [4].

Distant NM metastatic disease is uncommon compared with other forms of melanoma. Site of initial metastatic spread is more commonly the central nervous system (CNS) for NM than for melanomas that do not demonstrate neurotropism [9]. The reported rate of CNS metastases was 13% for DNM, 9% for DM, and 5% for a comparison melanoma group as may be expected with a neural invasive tumours. Another study of DNM reported 17.9% of 128 cases reviewed had distant metastases, with the lung being the most common site [4]. Other metastatic sites include brain, liver, skin, and bone [21, 23].

Neural extension or invasion can occur along large or small nerves, [6, 24] including cranial nerves, [2, 6, 7, 13, 18, 24, 25] most commonly the facial and trigeminal nerves [12, 18, 26]. Extended latent periods can exist between initial diagnosis and neural involvement that usually presents as a symptomatic neuropathy [7, 18, 25, 26]. Perineural spread of disease can occur beyond the skull base into Meckel's cave, the cavernous sinus and brain, and also expand in an antegrade fashion to include other cranial nerves [18]. An average latent period of 4.9 years (range 1.5–12 years) between initial diagnosis and recurrence into cranial nerves was reported in an eight-case respective study in 2004 [12, 18]. Presentation may include cranial nerve palsies, pain, numbness, difficulty with mastication, associated atrophy of jaw muscles, and the absence of corneal reflexes [7, 18, 25, 26]. One case of large nerve invasion outside of the head and neck involving the dorsal cutaneous branch of a spinal nerve has been reported [12]. Care must therefore be taken with patients who present with neuropathies where a previous suspicious lesion has been excised.

Patients may present with multiple local recurrences in the scar line of previously excised lesions likely due to inadequate excision [7, 17, 23]. Metastases may resemble their primaries or other forms of cutaneous melanoma [6]. Recurrence may occur as skip lesions, in areas particularly with perineural invasion where previous margins were clear [2]. Extensive neural spread can make cure difficult [19].

Initial presentation of NM, DNM, and DM tends to be at a more advanced stage compared with other melanoma subtypes [1, 2, 6, 7, 17]. Quinn et al. [12] reported that only 28% of patients presented with American Joint Committee on Cancer Stage I disease compared with the overall melanoma rate of 80%. Presence of neurotropism within a melanoma is an independent risk factor for local recurrence [1, 9, 12, 27, 28]. Local recurrence rate of 20% for DNM has been reported [12] occurring sooner than melanoma [21, 23]. However, overall DNM survival rates are better than for other melanoma [9, 12].

### 3.2. Investigation and Diagnosis

Diagnosis of NM, as with DM, can be difficult [5, 8, 14, 29, 30]. The list of differential diagnoses is extensive including both benign and malignant pathology. Delay in diagnosis can arise from a benign histopathological appearance [19]. Consideration of the diagnosis is key to early diagnosis and improved outcome [25].

A high index of suspicion and careful examination of skin, particularly in the head and neck region including the scalp and mucous membranes, is required for patients with potential melanocytic abnormalities. Particular attention needs to be taken in those with a previous history of melanoma to check for recurrence.

**Figure 1:**
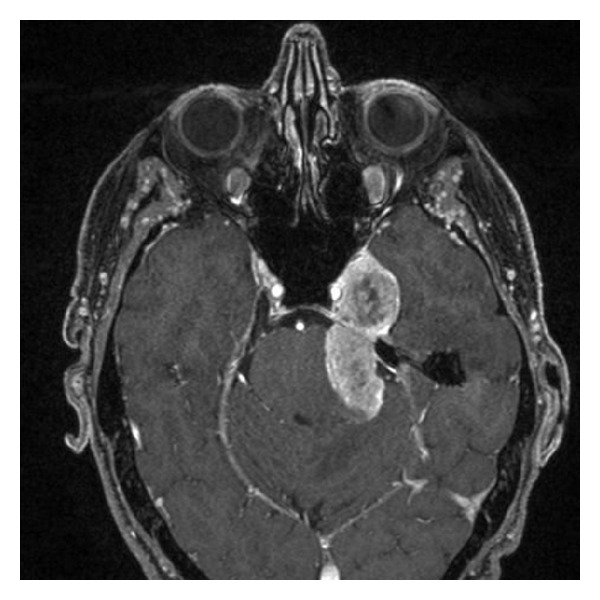
T1 Axial Fat Saturated, Gadolinium enhanced MRI scan of the head. This image displays the case of a 48-year-old man with a previous excision of a neurotropic melanoma of the face. Approximately one year from wide local excision he presented with facial pain and biopsy proven recurrence of neurotropic melanoma involving the mandibular division of the trigeminal nerve, filling the cavernous sinus and prepontine cistern with obvious brainstem compression.

Imaging is a key investigation for NM with suspected neural invasion. MRI can illustrate enhancement and thickening of neural structures or adjacent tissue planes, intracranial changes such as expansion or erosion of the skull base, mass in Meckel's cave, and bulging of the cavernous sinus which suggests perineural invasion as outlined in Figure 1 [7, 12, 18]. However, perineural enhancement on MRI offers multiple differential diagnoses including varying neoplastic, infectious, and inflammatory pathologies.

The use of FDG-PET scanning for diagnosis and staging NM has been little reported but the potential use exists, with the ability to demonstrate tumoural hypermetabolic activity associated with either the primary tumour, recurrence, or perineural spread [26].

For histopathological diagnosis, biopsy is required. This may be either excision of the entire lesion, open biopsy, core biopsy, or fine needle aspiration (FNA) biopsy of suspect lesions. As FNA biopsy of desmoplastic lesions has a low yield impairing diagnosis in NM [18] and has been associated with discrepant diagnostic opinions, [30] it is typically not recommended.

### 3.3. Histopathology

Diagnosis of DNM and NM can be a challenge for pathologists [2, 8, 14, 15, 30]. The histogeneses of NM, DNM, and DM are uncertain displaying both melanocytic and neuroectodermal properties and a variety of histogenic and immunophenotypic profiles [5, 6, 14]. Lesions may be associated with intraepidermal melanocytic proliferation (often lentigo maligna or superficial spreading melanoma) lesions but epithelioid and spindle cell components may also be involved [6, 14].

**Figure 2:**
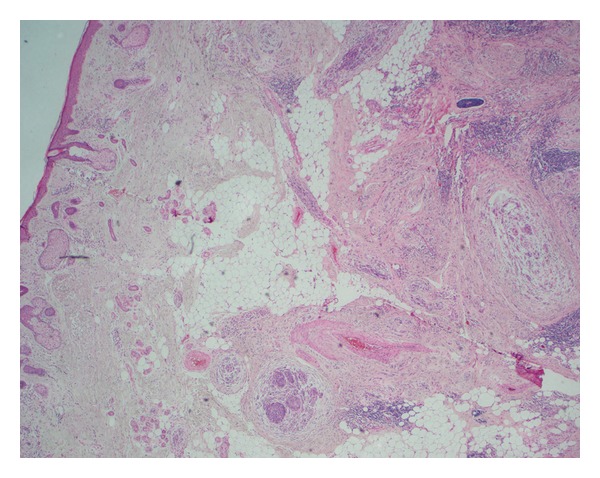
Low power (×20) view of skin with epidermis on the left and subcutis on the right. At this magnification we can identify nodules within the subcutis: at the centre, at the bottom, centre top, and on the right in the middle.

**Figure 3:**
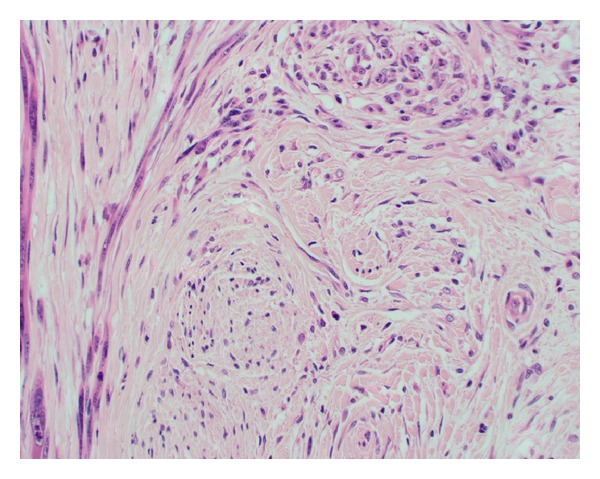
High power (×400) view of a nodule shows a nerve (bottom half) surrounded by melanoma (darker spindle cells on the left and top). The tumour cells at the top of the picture are arranged in a fasciculated (bundled) growth pattern, similar to the nerve next to it.

Microscopically NM is often poorly circumscribed, varying in size with extension into neural tissue, subcutaneous tissue, deep reticular layers, and fascia (Figure 2) [3]. The histopathological appearance is characteristically slender, hyperchromatic spindle cells and nuclei with mild to moderate amounts of cytological atypia, with or without neuroid differentiation [3, 5]. The majority of lesions are paucicellular. Vascular invasion is rare [5, 6]. Melanosomes may be difficult to locate and Schwannian differentiation may be present [19]. Neurotropic extension of spindle cells circumferentially around dermal nerves or thickened nerve tissue with abnormal cells in deeper tissue layers is distinct (Figure 3) [5]. Foci of neurotropism may be located beyond the margins of the main tumour.

Melanomas with neurotropism usually have a high Breslow thickness and advanced Clark's level (IV or V) at the time of presentation compared to other melanomas [3, 5, 9, 12, 17, 22]. Quinn et al. [12] reported that all DNMs were >1.5 mm thick in one of the largest DNM series published. Thickness within DNM lesion is a significant poor prognostic factor for overall survival. Difficulty in diagnosis may contribute to the reason for high Breslow and Clark's levels at presentation.

The mitotic rate is variable but more commonly low or intermediate in NM (0–4/mm^2^) [5, 9, 12]. However, high mitotic rate (i.e., >4/mm^2^) has been found to be a significant prognostic factor for time to recurrence and overall survival.

Immunophenotypically, NM and DNM also present a diagnostic dilemma, [30] lacking uniformity in expression of diagnostic antigens. Neurotropic Melanoma is almost uniformly positive for vimentin and S-100 [6, 15, 17] and less commonly positive for other melanocytic markers such as HMB-45, Melan-A, NKIC3, and Leu-7 [2, 8, 15, 19, 25]. However, S-100 is not specific for melanocytes and if the lesion is negative for S-100 it can make differentiation from other spindle cell lesions difficult. In addition, S-100 is common in neural tissue including neural tumours, chondrocytes, lipocytes, and dendritic cells which also can impede diagnosis [2, 5]. However, benefit may be conveyed in staining margins of resected lesions with S-100 to assess for adequate resection to assess the extent of invasion [5, 25]. Nerve growth factor receptor p75 has potential as an ancillary diagnostic stain to enable more reliable diagnosis [5, 15, 31].

There are currently no specific genetic assays for NM, DNM, or DM. However, frequent allelic loss at the neurofibromatosis type 1 (NF1) gene locus and high prevalence of basic fibroblast growth factor (bFGF) in cell nuclei offers potential of genetic assays and possible targeted gene therapy in the future [5].

### 3.4. Prognosis

Prognostic factors for cutaneous melanoma such as stage, tumour thickness, depth of invasion, and dermal mitotic rate apply to NM [5, 14, 17].

There are conflicting data as to the significance of neurotropism in melanoma as a poor prognostic factor for survival [9, 10, 17]. Baer et al. [9] reported 8-year survival for patients with DM as 90%, which decreased to 60% with the presence of neurotropism. The largest series thus far for DNM by Quinn et al. [12] reported significant increase in local recurrence when neurotropism was present but this had no significant impact on survival. In this series, DM and DNM 5-year survival was 72 and 58% at 10 years.

Poor prognosis in DNM and NM is associated with age, male gender, and head and neck locations of primary lesions, high Breslow thickness, close (<1 cm) or positive surgical margins. These are also the predictors of local recurrence [1, 4, 5, 12, 14, 28, 32]. 

While locally aggressive, DNM prognosis is better compared with similar staged conventional melanoma, emphasising the importance for local control [4]. Chen et al. [4] reported recurrence in 6.3% of DNM with a median time to local recurrence of 10 months. Local recurrence confers increased risk of further local recurrence and systemic metastases [3] which in turn is a poor prognostic marker [23]. Given the propensity for local recurrence, close, regular followup is recommended [21].

Perineural invasion into large nerves confers a poor prognosis, often altering the goal of treatment from cure to palliation [12, 18]. Untreated patients with intracranial metastases with neurological symptoms have a median survival of one month. Intracranial disease treated with radiotherapy can greatly extend median survival of patients by more than 8 months [7].

## 4. Management

### 4.1. Surgical

Depending on the size, site, and level of suspicion of a lesion, it may be initially biopsied or completely excised. However, once a diagnosis of DNM or NM is made, prompt wide local excision (WLE) with clear margins is the definitive management [3, 4, 12, 18, 23]. A minimum margin of 2 cm is recommended for NM to decrease the risk of local recurrence [1, 3, 5, 7, 12, 17]. Deep margins should include the fascia due to the invasive nature of the disease [23]. The need for extensive margins has meant that patients often require reresection [4, 6]. The location of the lesions particularly on the head and neck coupled with the invasive propensity can present problems to achieve reasonable margins whilst preserving function and cosmesis [2, 12, 21]. These factors and the benign appearance of some of these lesions may contribute to insufficient margins at time of initial resection. In areas such as the nose, there may be the need for local or free flaps to ensure sufficient margins are accomplished [21]. The increased morbidity and impact on quality of life of ongoing re-resection for local recurrence are important considerations in treatment [17].

For NM, there is a correlation between positive <1 cm or unknown margin status and local recurrence of DM [4, 17, 21, 23]. Given that neurotropism is associated with increased recurrence rates, nerves identified at surgery should be marked for specific histopathological analysis to examine presence of invasion [9].

The low incidence of nodal metastatic spread has meant that regional lymph node sampling and dissection are not routine when lesions are excised. Resections are undertaken when clinical palpable nodes are present and at the discretion of the surgeon [21]. Some argue that SLNB with lymphoscintigraphy is the most appropriate evaluation of nodal and micrometastatic spread to inform treatment decisionmaking [17, 23]. However, given a lack of research regarding SLNB in either NM or DM and the low yield of reported SLNB, the benefit of the procedure is still being debated [4, 5].

A tumour that has already demonstrated perineural spread, either on imaging or symptomatic neuropathy, may be unsuitable for definitive surgery and may require palliative radiotherapy or chemotherapy [12, 18].

### 4.2. Radiotherapy

Adjuvant radiotherapy after surgery is often considered particularly in “high-risk” regions like the head and neck where wide surgical margins are difficult to achieve. Currently, radiotherapy is considered in the postoperative setting for recurrent lesions or where margins are positive or deemed inadequate [4, 7, 20]. Radiotherapy as definitive treatment for inoperable disease has been shown to provide local control of disease [20]. In this case the radiotherapy volume should encompass all of the gross disease (including the primary site), the named nerve extending to the skull base, and neural foramina and any evidence of intracranial disease [2, 4]. In many cases, however, those with inoperable disease are incurable and radiotherapy is effective in the palliation of these patients.

Limited data are available for the use of only radiotherapy for treatment of NM. Chen et al. [4] reported on 128 cases of DNM, of which 21% underwent adjuvant radiotherapy. The authors found that those patients who underwent radiotherapy had more advanced tumours and less adequate surgical margins.

Foote et al. [1] reported on 24 patients that received radiotherapy for DM including DNM. The median dose was 48 Gy in 20 fractions over 4 weeks (dose range 48–60 Gy, fraction range 20–30). The 3-year in-field relapse-free survival was 91%, 3-year relapse-free survival was 86%, and 3-year overall survival was 83% [1].

No prospective randomised trials to date have evaluated radiotherapy preventing local recurrence following surgical excision. A randomised trial of postoperative radiation treatment following wide excision of neurotropic melanoma of the head and neck is currently underway with the Trans Tasman Radiation Oncology Group (TROG 08.09)/Australia and New Zealand Melanoma Trials Group (ANZMTG 01.09).

### 4.3. Chemotherapy

Chemotherapy is not routinely offered for treatment of localised NM. It has been utilised for patients with hematogenously disseminated disease. For disseminated disease palliative chemotherapy regimens have included use of chemotherapy and immunotherapy agents such as dacarbazine (DTIC), ipilimumab (IPI), interferon-*α*, aldesleukin, and BRAF inhibitors such as vemurafenib (in patients with BRAF-mutated melanoma). There have been no dedicated studies regarding efficacy of chemotherapy treatments in disseminated NM or DNM disease [2, 7].

## 5. Conclusion

Neurotropic melanoma is a rare but locally aggressive variant of melanoma. It is varied and often benign appearance makes diagnosis challenging and may contribute to advanced stage at presentation and propensity of local recurrence. Low rates of nodal and distant metastatic disease highlight the importance of local control. This is achieved through excision of lesion with wide margins of at least 2 cm and adjuvant radiotherapy where indicated. Potential exists for clarification in diagnostic criteria for NM and evaluation of adjuvant radiotherapy.

## Abbreviations

CNS: Central nervous system
DM: Desmoplastic melanoma
DNM: Desmoplastic neurotropic melanoma
IPI: Ipilimumab
NM: Neurotropic melanoma
MRI: Magnetic Resonance Imaging
SLNB: Sentinel lymph node biopsy
TROG: Trans Tasman radiation oncology group.
